# Low Serum Magnesium Levels Are Associated With Hemorrhagic Transformation After Mechanical Thrombectomy in Patients With Acute Ischemic Stroke

**DOI:** 10.3389/fneur.2022.831232

**Published:** 2022-03-23

**Authors:** Huijia Qiu, Rui Shen, Liuwei Chen, Sajan Pandey, Jiping Sun, Haoyu Deng

**Affiliations:** ^1^Department of Neurosurgery, Shanghai Tenth People's Hospital, Tongji University, Shanghai, China; ^2^Department of Medicine, Faculty of Medicine, University of British Columbia, Vancouver, BC, Canada; ^3^Centre for Heart and Lung Innovation, St.Paul's Hospital, University of British Columbia, Vancouver, BC, Canada; ^4^Department of Vascular Surgery, Renji Hospital, School of Medicine, Shanghai Jiaotong University, Shanghai, China

**Keywords:** magnesium, hemorrhagic transformation, mechanical thrombectomy, acute ischemic stroke, risk

## Abstract

**Objective:**

In patients with acute ischemic stroke (AIS), hemorrhagic transformation (HT) is a major complication after mechanical thrombectomy (MT). This study aimed to investigate the relationship between serum magnesium levels and HT after MT.

**Methods:**

We collected 199 cases of consecutive AIS that received MT due to acute anterior circulation occlusions in our institution between January 2017 and January 2020. Baseline serum magnesium was obtained from all patients on admission before MT. The patients were divided into two groups based on the occurrence of HT. Univariate and multivariate analyses were performed to investigate whether magnesium was an independent predictor of HT. The receiver operating characteristic (ROC) curve and area under the curve (AUC) were determined.

**Results:**

Of the 199 enrolled patients, 40 (20.1%) presented with HT, and 12 (6%) developed symptomatic intracranial hemorrhage (sICH). Patients with HT had lower serum magnesium levels compared to those without HT (0.76 [0.69–0.80] vs. 0.84 [0.80–0.90], *p* < 0.001). The multivariate logistic analysis showed that the serum magnesium level (odds ratio, [OR]: 0.000, 95% confidence interval [CI]: 0.000–0.001, *p* < 0.001) was significantly associated with the occurrence of HT. The ROC curve analysis revealed that the serum magnesium level could predict HT with an AUC of.820 (95% CI: 0.750–0.891 *p* < 0.001). Serum magnesium ≤ 0.80 mmol/L could predict HT with a sensitivity of 79.2% and a specificity of 70.0%. Of interest, the serum magnesium level was not associated with HT when the baseline of serum magnesium was higher than the cut-off value (0.80 mmol/L) in the subgroup analysis.

**Conclusions:**

Lower baseline serum magnesium levels (<0.80 mmol/L) on admission are associated with increased risk of HT in AIS patients receiving MT.

## Introduction

The treatment choice for acute ischemic stroke (AIS) has been improving, from early administration of intravenous recombinant tissue plasminogen activator (rtPA) to mechanical thrombectomy (MT) or a combination of both (1). Hemorrhagic transformation (HT) frequently occurs after AIS. Whereas the symptomatic form of transformation has been reported as low as 0.6% to maximum up to 20% (2, 3), and it can contribute to higher mortality and morbidity rates after a stroke (4, 5). Thus, the identification of the risk factors for HT is of utmost importance for patients receiving MT.

Preclinical models of stroke have shown Magnesium (Mg) as a neuroprotective agent (6). It can maintain the integrity of microvascular endothelial barriers *via* anti-oxidation and anti-inflammatory regulations (7). Mg is also involved in the coagulation cascade and platelet activation (8–10), and its deficiency can dysregulate the coagulation system. The relationships between serum Mg (sMg) and functional outcome endpoints in patients with AIS have been extensively studied. However, the Field administration of stroke therapy-Mg (FAST-MAG) and intravenous Mg efficacy in stroke (IMAGE) trials did not show improved outcomes with early intravenous Mg sulfate (11, 12). However, other emerging studies suggest its role in hemorrhagic transformation (13, 14). A study done by Cheng et al. shows that low sMg levels are related to hemorrhagic transformation following thrombolysis in AIS (14), but there is no study revealing its association with patients undergoing mechanical embolectomy. The present research aimed to investigate if a low sMg level at admission could predict HT among patients with AIS who received MT.

## Methods

### Patients

Data from patients with AIS who received MT due to acute anterior circulation occlusions in our institution from January 2017 to January 2020 were acquired and subjected to further analysis.

The inclusion criteria were listed as follows: (i) diagnosed AIS and admitted within 6 h after an ictus; (ii) occlusion of the internal carotid artery or the M1/M2 segment of the middle cerebral artery confirmed by CT angiography and/or digital subtraction angiography; (iii) patients who had undergone mechanical thrombectomy; (vi) aged ≥18.

The following exclusion criteria were listed as follows: (i) posterior circulation occlusion; (ii) patients with prior systemic diseases, including severe liver and kidney dysfunction, severe hematological diseases, and malignant tumors; (iii) lack of data of sMg levels on admission or subsequent cranial CT scans. Ethical approval for this research was obtained from the Ethics Committees of Shanghai Tenth people's hospital.

### Endovascular Procedure

Patients received MT in compliance with the recommendations of the American Stroke Association/American Heart Association (15). MT was conducted alone or combined with intravenous thrombolysis, at <6 h following the first symptom onset. A solitaire stent retriever in combination with the intracranial support aspiration catheter was used for the MT SolitaireFR with intracranial support catheter for mechanical thrombectomy (SWIM) approach. The detailed methods for SWIM were reported previously (16). For anesthesia, we preferred conscious sedation, with general anesthesia reserved only when necessary. For tandem occlusion stroke, intracranial occlusion was treated first whenever possible. For underlying stenosis or dissection, rescue stenting and/or balloon angioplasty was performed at the treating physician's discretion.

### Data Acquisition

Clinical and baseline information was acquired from the patients, including age, gender, vascular risk factors (e.g., hyperlipidemia, hypertension, diabetes mellitus, atrial fibrillation, coronary artery disease, current smoking, history of stroke), National Institutes of Health Stroke Scale (NIHSS) score at admission, application of intravenous thrombolysis, procedure duration, number of passes of retriever, thrombus location, onset-to-treatment time, stroke etiology, recanalization status, systolic and diastolic blood pressure at admission, and lab findings at admission (Blood glucose level, sMg level, platelet count, the international normalized ratio [INR], activated partial thromboplastin time [APTT], and prothrombin time [PT]). Stroke etiology was established according to the Trial of Org 10172 in Acute Stroke (TOAST) criteria (17). The recanalization status was evaluated based on the Thrombolysis In Cerebral Infarction (TICI) score, and the recanalization was considered to be successful when the TICI score was 2b or 3 (18). The blood sample was withdrawn from each patient on admission before mechanical thrombectomy. All specimens were analyzed within 1 h following venipuncture. Laboratory parameters, such as sMg, were assessed at the local laboratories. Routine CT scanning was performed at 24–36 h after MT, and then on a case-by-case basis in case of neurological deterioration to detect HT.

### Assessment of Hemorrhagic Transformation

Computed tomography (CT) images were independently reviewed in a randomized order by two experienced interventional neuroradiologists blinded to clinical conditions. Afterward, consensus readings were conducted to obtain a reference standard for statistical analysis. The outcome measures were the occurrence of HT within 48 h after MT. Based on the classification described in the European cooperative acute stroke study (ECASS), the hemorrhagic transformation was categorized into parenchymal hemorrhage (PH) and hemorrhagic infarction (HI) (19). Symptomatic intracerebral hemorrhage (sICH) was diagnosed based on the Heidelberg Bleeding Classification (20). HT associated with the procedure itself, such as subarachnoid hemorrhage, arterial dissection, or secondary to vessel perforation, was ruled out in our analysis.

### Statistical Analyses

The program SPSS v26.0 (SPSS v26.0, IBM, Armonk, NY, USA) was used to perform the statistical tests. A categorical variable was presented as a number (frequency), while a continuous variable was expressed as a median (interquartile range [IQR]). In the univariate analysis, a categorical variable was compared by the χ^2^ test or Fisher's exact test, whereas the continuous variable was compared by the Mann-Whitney U test. All significant variables (*p* < 0.10) in the univariate analysis were chosen for multivariate regression analysis. The association between sMg level and HT was examined with the multivariate logistic regression model. The receiver operating characteristic (ROC) curves were drawn to evaluate the overall discrimination ability of sMg level for predicting HT. The optimum cut-off value was established using the Youden index. A two-tailed value of *p* < 0.05 was deemed statistically significant.

## Results

In total, 220 patients with AIS who underwent MT due to anterior circulation occlusions were screened for eligibility. Two patients were excluded due to the absence of follow-up imaging; 17 patients were excluded owing to incomplete laboratory data; additional two patients were excluded due to procedure-related hemorrhage (secondary to vessel perforation). Finally, 199 patients with AIS were ultimately included, with a median age of 69 years (IQR: 64–79). Of them, 107 (53.8%) patients were female. The median NIHSS score at admission was 13 with IQR between 10 and 15. In addition, 176 (88.4%) patients had successful recanalization (TICI ≥2b). The median sMg level was 0.82 mmol/L with an IQR between 0.78–0.89. Table 1 shows the baseline and clinical information.

Among the 199 patients, 40 (20.1%) manifested with HT, including HI (*n* = 17) and PH (*n* = 23). Of these 40 patients, 12 (30%) developed sICH. These patients were divided into two groups depending on the occurrence of HT. Univariate analysis showed that patients with HT had lower serum Mg levels on admission compared to those without HT (0.76 [0.69–0.80] vs.0.84 [0.80–0.90], *p* < 0.001). Patients with HT also exhibited a higher risk of diabetes mellitus (*p* = 0.012), a higher INR (*p* = 0.026), and PT (*p* = 0.003) on admission compared to non-HT patients (Table 1). The multivariate regression data indicated that sMg level (odds ratio, [OR]: 0.000, 95% CI: 0.000–0.001, *p* < 0.001) and PT (OR: 2.251, 95% CI: 1.075–4.715, *p* = 0.031) on admission were remarkably associated with the occurrence of HT (Table 2).

**Table 1:** Univariate analysis for predictors of hemorrhagic transformation after mechanical thrombectomy.

**Variable**	**Total patients**	**HT**		***P value***
	**(*n* = 199)**	**Yes (*n* = 40)**	**No (*n* = 159)**	
Age (years)	69 (64-79)	69 (65-76)	69 (63-80)	0.972
Sex (Female)	107 (53.8%)	21 (52.5%)	86 (54.1%)	0.857
Hypertension	145 (72.9%)	33 (82.5%)	112 (70.4%)	0.125
Diabetes mellitus	49 (24.6%)	16 (40.0%)	33 (20.8%)	**0.012**
Hyperlipidemia	34 (17.1%)	4 (10.0%)	30 (18.9%)	0.183
Atrial fibrillation	85 (42.7%)	21 (52.5%)	64 (40.3%)	0.162
Coronary artery disease	41 (20.6%)	6 (15.0%)	35 (22.0%)	0.327
Smoking	52 (26.1%)	7 (17.5%)	45 (28.3%)	0.165
Previous stroke	59 (29.6%)	13 (32.5%)	46 (28.9%)	0.659
NIHSS score on admission	13 (10-15)	13 (10-16)	13 (10-15)	0.329
Intravenous thrombolysis	118 (59.3%)	25 (62.5%)	93 (58.5%)	0.645
Time to procedure	190 (165-270)	180 (158-253)	190 (165-275)	0.857
Procedure duration	45 (30-70)	45 (30-75)	50 (30-70)	0.884
Passes of retriever	2 (1-3)	2 (1-3)	2 (1-3)	0.767
Thrombus location				0.266
Intracranial ICA	90 (45.2%)	22 (55.0%)	68 (42.8%)	
M1 MCA segment	96 (48.2%)	17 (42.5%)	79 (49.7%)	
M2 MCA segment	13 (6.5%)	1 (2.5%)	12 (7.5%)	
Stroke subtype				0.401
Cardioembolism	88 (44.2%)	21 (52.5%)	67 (42.1%)	
Large artery atherosclerosis	68 (34.2%)	13 (32.5%)	55 (34.6%)	
Undetermined etiology	43 (21.6%)	6 (15.0%)	37 (23.3%)	
Successful recanalization (TICI≥2b)	176 (88.4%)	36 (90.0%)	140 (88.1%)	0.730
Systolic BP at admission	142 (129-156)	140 (129-157)	143 (129-156)	0.916
Diastolic BP at admission	80 (71-90)	82 (72-93)	80 (70-88)	0.245
Laboratory finding				
Glucose at admission	7.3 (6.3-9.1)	7.5 (6.4-9.4)	7.2 (6.1-9.0)	0.259
Serum magnesium	0.82 (0.78-0.89)	0.76 (0.69-0.80)	0.84 (0.80-0.90)	**<0.001**
Platelet count	176 (146-218)	166 (123-223)	179 (150-216)	0.264
INR	1.08 (1.03-1.16)	1.13 (1.05-1.26)	1.07 (1.02-1.15)	**0.026**
PT	12.3 (11.6-13.3)	12.9 (12.2-13.5)	12.2 (11.5-13.1)	**0.003**
APTT	27.6 (26.1-29.9)	28.3 (26.2-30.0)	27.5 (26.0-29.8)	0.431

*Data are n (%), or median (interquartile). Univariate analysis showed that patients with HT had lower serum Mg levels on admission compared to those without HT (0.76 [0.69–0.80] vs.0.84 [0.80–0.90], p <0.001). Patients with HT also exhibited a higher risk of diabetes mellitus (p = 0.012), a higher INR (p = 0.026), and PT (p = 0.003) on admission compared to non-HT patients are in bold*.

**Table 2:** Multivariate analysis for predictors of hemorrhagic transformation after mechanical thrombectomy.

**Predictors**	**OR (95% CI)**	***P Value***
Diabetes mellitus	2.400 (0.983-5.861)	0.055
Serum magnesium	0.000 (0.000-0.001)	**<0.001**
INR	0.002 (0.000-4.479)	0.112
PT	2.251 (1.075-4.715)	**0.031**

*OR, odds ratio; CI, confidence interval. The multivariate regression data indicated that sMg level (odds ratio, [OR]: 0.000, 95% CI: 0.000–0.001, p <0.001) and PT (OR: 2.251, 95% CI: 1.075–4.715, p = 0.031) on admission were remarkably associated with the occurrence of HT are in bold*.

**Figure 1:**
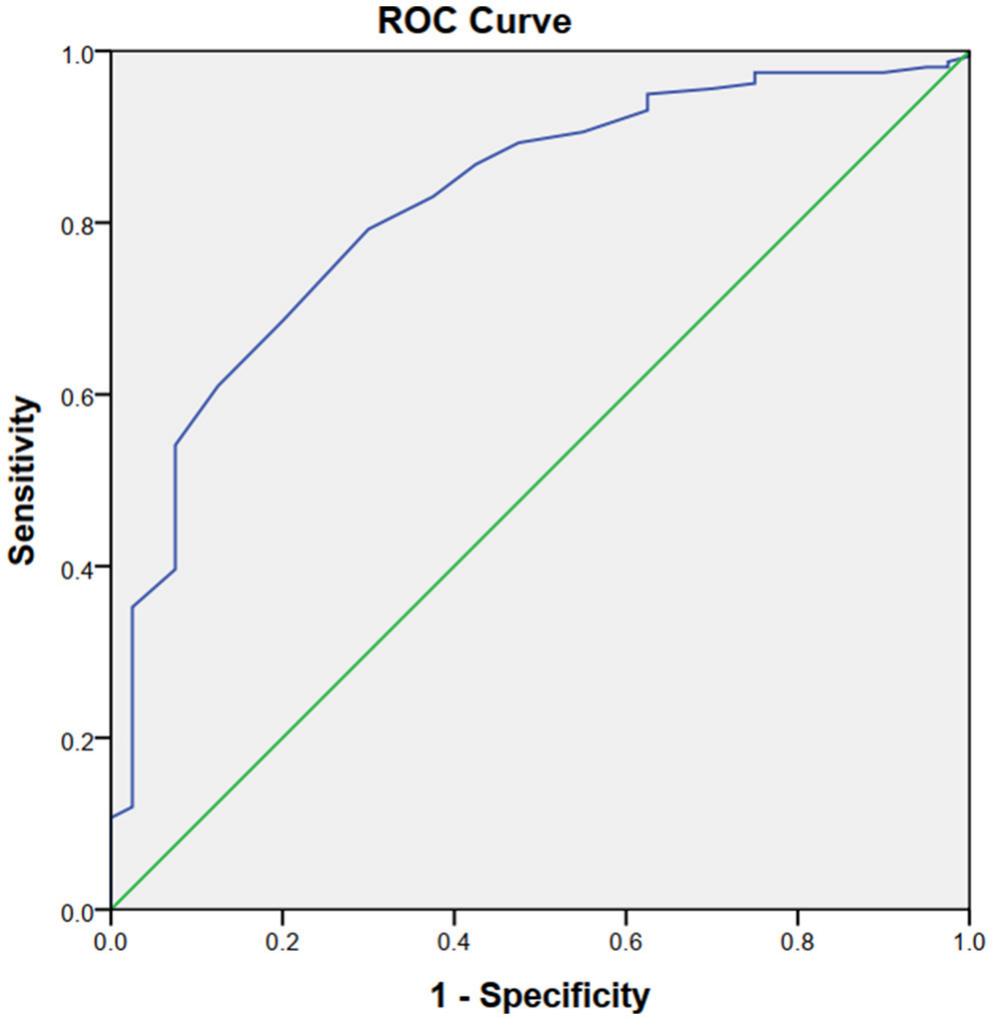
The ROC curves demonstrated that the sMg level could estimate HT risk with an area under the curve (AUC) of 0.820 Q21 (95% CI: 0.750–0.891 *p* < 0.001).

The ROC curves demonstrated that the sMg level could estimate HT risk with an area under the curve (AUC) of 0.820 (95% CI: 0.750–0.891 *p* < 0.001, Figure 1). The optimum cut-off value for Mg to HT was 0.80 mmol/L. It was found that HT could be predicted by sMg ≤ 0.80 mmol/L, with the sensitivity and specificity of 79.2 and 70.0%, respectively.

After stratification of patients according to the cut-off value, the sMg levels (<0.80 mmol/L) were associated with HT risk (OR: 0.000, 95% CI: 0.000–0.520, *p* = 0.036). However, no obvious relationship was found between the sMg levels (>0.80 mmol/L) and HT risk (OR: 0.000, 95% CI: 0.000–1.617, *p* = 0.056; Table 3).

**Table 3:** Stratified logistic regression analysis to identify relationships between serum magnesium levels and risk of hemorrhagic transformation after mechanical thrombectomy.

**Serum magnesium**	**OR (95% CI)**	***P Value***
≤ cut-off value ( ≤ 0.80 mmol/L)	0.000 (0.000-0.520)	0.036
> cut-off value (>0.80 mmol/L)	0.000 (0.000-1.617)	0.056

*The model was adjusted for the same confounding factors n Table 2*.

Among the 40 patients with HT, 17 presented with HI, and 23 presented with PH; 12 were sICH, and 28 were asymptomatic ICH. There was no difference in sMg level between PH and HI (0.75 [0.69–0.80] vs. 0.77 [0.70–0.80], *p* = 0.533), sICH and asymptomatic ICH (0.73 [0.67–0.79] vs. 0.76 [0.73–0.80], *p* = 0.328).

## Discussion

In this retrospective study, the relationship between sMg levels and the risk of HT in patients with AIS following MT was evaluated. Our study observed that patients with HT had lower sMg levels than non-HT patients. Multivariate analysis indicated that a low sMg level could be a significant risk factor for HT. The ROC curves showed that a serum Mg level ≤ 0.80 mmol/L could estimate HT risk with the sensitivity and specificity of 79.2 and 70.0%, respectively. However, the negative relationship between Mg and HT did not appear to hold when sMg levels were higher than the cut-off value (0.80 mmol/L). Consequently, a low serum Mg level at admission could predict HT in patients with AIS who had undergone MT.

Magnesium (Mg) is a very important micromineral in the diet with various roles in the human body. It is associated with platelet activation and coagulation cascade (8–10). Meanwhile, it is also an abundant endogenous neuroprotective agent closely associated with AIS (21). It can maintain the integrity of the microvascular endothelial barriers *via* anti-oxidation and anti-inflammatory regulations (22). Mg deficiency can lead to deleterious effects on endothelium integrity. The activation of ischemia-induced BBB breakdown is mainly caused by endothelial damage (23). Previous research has demonstrated the clinical significance of Mg in AIS. One meta-analysis found that Mg intake level was inversely related to the risk of AIS (24). A decreased sMg level at admission could be a significant risk factor for in-hospital mortality in patients with AIS (25). Tan et al. enrolled 1,212 patients with AIS and found that low sMg levels were remarkably associated with HT risk in patients with AIS (13). Similar to Tan's study, Cheng's study enrolled 242 patients with AIS following intravenous thrombolysis therapy, from which they found that low sMg levels demonstrated an increased risk of HT after intravenous thrombolysis (14). To our knowledge, our study is the first to report the association between Mg and HT in patients with AIS treated with MT.

The relationship between low serum Mg and HT after MT is still unclear. Mg is required to maintain human endothelial integrity. High Mg has been proved to inhibit glutamate release, block glutamate receptors, and improve mitochondrial calcium buffering and cellular energy metabolism, displaying a glioprotective and neuroprotective effect on penumbra in AIS (26–29). Conversely, low Mg will initiate an inflammatory cascade, disrupt the BBB integrity, and damage the vascular endothelium (7). The bleeding tendency would increase once the BBB loses its integrity. Collectively, our study hypothesizes that low Mg aggravates the disruption of a neurovascular unit, which is commonly accepted as a potential risk factor for HT. A low sMg level leads to coagulation dysfunction *via* its involvement through coagulation cascade and platelet activation, which may serve as an additive effect on compromised BBB (8–10). Mg can accelerate the initiation of factor X *via* the tissue factor-, factor VIIa- and factor IXa-mediated pathways and shorten the prothrombin time (9, 30). It also promotes platelet adhesion onto collagen, independent of platelet aggregation and release (10). In patients with subarachnoid or intracerebral hemorrhage, the hemostatic properties of Mg have been found, as well as the negative association between sMg levels and hematoma volume (31, 32). Besides, arterial stiffness is identified as a significant risk factor for HT in AIS (33). Mg is closely related to arterial stiffness. A recent study shows that arterial stiffness can be alleviated by prolonged supplementation with Mg in particular patients (34).

Our study also found that when the sMg concentration is higher than 0.80 mmol/L, the effect of Mg on HT after MT is no more active, which indicates the specific threshold of Mg to reduce the risk of HT. Mg can maintain the integrity of the endothelium at physiological levels, but excessive Mg improves the integrity (35). As a result, Mg may offer better BBB protection in a particular concentration range. In addition, this study also found that a high Mg level may inversely decrease platelet activities and increase bleeding time (9, 36). Our results are in good agreement with these findings, which suggest that the risk of HT may not alleviate after the sMg level exceeds a specific threshold (0.80 mmol/L in our research). But we did not find a significant difference in sMg levels among HI, PH, sICH, and asymptomatic ICH. We speculate that the negative results were due to our small sample size. Additional studies are warranted to confirm the actual relationships between sMg and type of HT.

Our findings imply that sMg can predict a person's vulnerability to HT after MT and identify those at high risk. Supplementation with Mg can decrease the risk of HT in patients receiving MT, at least to a certain extent. On the contrary, the FAST-MAG and IMAGES trials indicated that MgSO4 therapy might not improve prognosis in patients with AIS (11, 12). It is likely because it is difficult for Mg to transport across the BBB, thereby reducing its accumulation in the brain tissue. But the limitation of these studies is that the case enrollment includes both ischemic and hemorrhagic stroke patients. None of the patients with ischemic stroke had undergone MT, and all patients were randomly given intravenous Mg supplementation without considering their baseline sMg concentrations. Thus, further research is warranted to examine the effect of Mg supplementation on the prognosis of patients with AIS with low sMg levels in the era of MT.

There are a few limitations that need to be addressed. First, this study was a retrospective study conducted at a single center. As a result, the conclusion may be biased and not appliable to the whole population. Second, we only measured the total Mg instead of Mg ions that exhibit a direct physiological role. The association between sMg and type of HT remains unclear. Third, the data is not complete, clinical information such as infarct regions, the history of anticoagulants, and antiplatelet drugs, were missing in the current study. Fourth, we did not collect data on the extent of ischemic change, for example, the Alberta Stroke Program Early Computed Tomography Score (ASPECTS) on admission and the infarct volume due to the incomplete clinical data and images. Hence, more studies are needed to verify the impacts of Mg on HT.

## Conclusions

Our study reveals that a low baseline sMg level on admission is related to HT risk in patients with AIS receiving MT, especially at the cut-off value of 0.80 mmol/L. Therefore, sMg can be applied in the clinical setting to identify patients at high risk of HT.

## Data Availability Statement

The original contributions presented in the study are included in the article/supplementary material, further inquiries can be directed to the corresponding author/s.

## Ethics Statement

The studies involving human participants were reviewed and approved by Shanghai Tenth People's Hospital, Tongji University. The patients/participants provided their written informed consent to participate in this study.

## Author Contributions

HD and JS were responsible for the study of design, data analysis, and drafting of the manuscript. HQ and RS were responsible for sample preparation, patients' care, and took part in data analysis. SP and LC took part in patient care, establishing laboratory techniques, and data interpretation. All authors contributed to the article and approved the submitted version.

## Funding

This research was supported by the Fundamental Research Funds for the Central Universities No. 22120180047.

## Conflict of Interest

The authors declare that the research was conducted in the absence of any commercial or financial relationships that could be construed as a potential conflict of interest.

## Publisher's Note

All claims expressed in this article are solely those of the authors and do not necessarily represent those of their affiliated organizations, or those of the publisher, the editors and the reviewers. Any product that may be evaluated in this article, or claim that may be made by its manufacturer, is not guaranteed or endorsed by the publisher.

## References

[1] Powers WJ Rabinstein AA Ackerson T Adeoye OM Bambakidis NC Becker K. Guidelines for the early management of patients with acute ischemic stroke: 2019 update to the 2018 guidelines for the early management of acute ischemic stroke: a guideline for healthcare professionals from the American heart association/American stroke association. Stroke. (2019) 50:e344–e418. 10.1161/STR.0000000000000211 31662037

[2] Jaillard A Cornu C Durieux A Moulin T Boutitie F Lees KR. Hemorrhagic transformation in acute ischemic stroke: the mast-e study. Stroke. (1999) 30:1326–32. 10.1161/01.STR.30.7.1326 10390303

[3] Bang OY Saver JL Kim SJ Kim G-M Chung C-S Ovbiagele B. Collateral flow averts hemorrhagic transformation after endovascular therapy for acute ischemic stroke. Stroke. (2011) 42:2235–9. 10.1161/STROKEAHA.110.604603 21737798

[4] Fiorelli M Bastianello S von Kummer R Del Zoppo GJ Larrue V Lesaffre E. Hemorrhagic transformation within 36 hours of a cerebral infarct: relationships with early clinical deterioration and 3-month outcome in the european cooperative acute stroke study I (Ecass I) cohort. Stroke. (1999) 30:2280–4. 10.1161/01.STR.30.11.2280 10548658

[5] Khatri P Wechsler LR Broderick JP. Intracranial hemorrhage associated with revascularization therapies. Stroke. (2007) 38:431–40. 10.1161/01.STR.0000254524.23708.c9 17234988

[6] Saver JL Starkman S Eckstein M Stratton SJ Pratt FD Hamilton S. Prehospital use of magnesium sulfate as neuroprotection in acute stroke. New Engl J Med. (2015) 372:528–36. 10.1056/NEJMoa1408827 25651247 PMC4920545

[7] Wolf FI Trapani V Simonacci M Ferré S Maier JA. Magnesium deficiency and endothelial dysfunction: is oxidative stress involved? Magnesium Res. (2008) 21:58–64. 18557135

[8] Messer AS Velander WH Bajaj SP. Contribution of magnesium in binding of factor ixa to the phospholipid surface: implications for vitamin k-dependent coagulation proteins. J Thrombosis Haemostasis: JTH. (2009) 7:2151. 10.1111/j.1538-7836.2009.03634.x 19817987 PMC2885445

[9] Van den Besselaar A. Magnesium and manganese ions accelerate tissue factor-induced coagulation independently of factor Ix. Blood Coagulat Fibrinolysis. (2002) 13:19–23. 10.1097/00001721-200201000-00003 11994563

[10] Santoro SA. Identification of a 160,000 dalton platelet membrane protein that mediates the initial divalent cation-dependent adhesion of platelets to collagen. Cell. (1986) 46:913–20. 10.1016/0092-8674(86)90073-5 3757029

[11] Muir KW Lees KR Ford I Davis S. Magnesium for acute stroke (intravenous magnesium efficacy in stroke trial): randomised controlled trial. Lancet (London, England). (2004) 363:439–45. 10.1016/S0140-6736(04)15490-1 14962524

[12] Shkirkova K Starkman S Sanossian N Eckstein M Stratton S Pratt F. Paramedic initiation of neuroprotective agent infusions: successful achievement of target blood levels and attained level effect on clinical outcomes in the fast-mag pivotal trial (field administration of stroke therapy - magnesium). Stroke. (2017) 48:1901–7. 10.1161/STROKEAHA.116.015664 28583999

[13] Tan G Yuan R Wei C Xu M Liu M. Serum magnesium but not calcium was associated with hemorrhagic transformation in stroke overall and stroke subtypes: a case-control study in China. Neurologic Sci : official J Italian Neurologic Soc Italian Soc Clinic Neurophysiol. (2018) 39:1437–43. 10.1007/s10072-018-3445-8 29804167

[14] Cheng Z Huang X Muse FM Xia L Zhan Z Lin X. Low serum magnesium levels are associated with hemorrhagic transformation after thrombolysis in acute ischemic stroke. Front Neurol. (2020) 11:962. 10.3389/fneur.2020.00962 32982953 PMC7492199

[15] Powers WJ Derdeyn CP Biller J Coffey CS Hoh BL Jauch EC. 2American heart association/American stroke association focused update of the 2013 guidelines for the early management of patients with acute ischemic stroke regarding endovascular treatment: a guideline for healthcare professionals from the American Heart Association/American stroke association. Stroke. (2015) 46(10):3020–35. 10.1161/STR.0000000000000074 26123479

[16] Chen L Shen R Zhang X Chen Z Lu H Zhou X. A single-center comparative study of the swim technique in the treatment of acute ischemic stroke due to anterior circulation occlusion. Thrombosis Res. (2020) 192:131–3. 10.1016/j.thromres.2020.05.011 32474253

[17] Adams HPJ Bendixen BH Kappelle LJ Biller J Love BB Gordon DL. Classification of subtype of acute ischemic stroke. definitions for use in a multicenter clinical trial. toast. trial of org 10172 in acute stroke treatment. Stroke. (1993) 24:35–41. 10.1161/01.STR.24.1.35 7678184

[18] Higashida RT Furlan AJ Roberts H Tomsick T Connors B Barr J. Trial design and reporting standards for intra-arterial cerebral thrombolysis for acute ischemic stroke. Stroke. (2003) 34:e109–37. 10.1161/01.STR.0000082721.62796.09 12869717

[19] Hacke W Kaste M Fieschi C Toni D Lesaffre E von Kummer R. Intravenous thrombolysis with recombinant tissue plasminogen activator for acute hemispheric stroke. JAMA. (1995) 274:1017–25. 10.1001/jama.1995.03530130023023 7563451

[20] von Kummer R Broderick JP Campbell BCV Demchuk A Goyal M Hill MD. The heidelberg bleeding classification: classification of bleeding events after ischemic stroke and reperfusion therapy. Stroke. (2015) 46:2981–6. 10.1161/STROKEAHA.115.010049 26330447

[21] Kirkland AE Sarlo GL Holton KF. The role of magnesium in neurological disorders. Nutrients. (2018) 10:6. 10.3390/nu10060730 29882776 PMC6024559

[22] Zhu D You J Zhao N Xu H. Magnesium regulates endothelial barrier functions through Trpm7, Magt1, and S1p1. Adv Sci. (2019) 6:1901166. 10.1002/advs.201901166 31559137 PMC6755513

[23] Krueger M Bechmann I Immig K Reichenbach A Härtig W Michalski D. Blood-Brain barrier breakdown involves four distinct stages of vascular damage in various models of experimental focal cerebral ischemia. J Cerebr Blood Flow Metabol: Offic J Int Soc Cerebr Blood Flow Metabol. (2015) 35:292–303. 10.1038/jcbfm.2014.199 25425076 PMC4426746

[24] Larsson SC Orsini N Wolk A. Dietary magnesium intake and risk of stroke: a meta-analysis of prospective studies. Am J Clinic Nutri. (2012) 95:362–6. 10.3945/ajcn.111.022376 22205313

[25] You S Zhong C Du H Zhang Y Zheng D Wang X. Admission low magnesium level is associated with in-hospital mortality in acute ischemic stroke patients. Cerebrovascul Dis. (2017) 44:35–42. 10.1159/000471858 28419989

[26] Lin J-Y Chung S-Y Lin M-C Cheng F-C. Effects of magnesium sulfate on energy metabolites and glutamate in the cortex during focal cerebral ischemia and reperfusion in the gerbil monitored by a dual-probe microdialysis technique. Life Sci. (2002) 71:803–11. 10.1016/S0024-3205(02)01738-1 12074939

[27] Nowak L Bregestovski P Ascher P Herbet A Prochiantz A. Magnesium gates glutamate-activated channels in mouse central neurones. Nature. (1984) 307:462–5. 10.1038/307462a0 6320006

[28] Smith DA Connick JH Stone TW. Effect of changing extracellular levels of magnesium on spontaneous activity and glutamate release in the mouse neocortical slice. Br J Pharmacol. (1989) 97:475–82. 10.1111/j.1476-5381.1989.tb11975.x 2758226 PMC1854541

[29] Favaron M Bernardi P. Tissue-specific modulation of the mitochondrial calcium uniporter by magnesium ions. FEBS Lett. (1985) 183:260–4. 10.1016/0014-5793(85)80789-4 3987891

[30] van den Besselaar AM van Dam W Sturk A Bertina RM. Prothrombin time ratio is reduced by magnesium contamination in evacuated blood collection tubes. Thrombosis and haemostasis. (2001) 85:647–50. 10.1055/s-0037-1615647 11341499

[31] Liotta EM Prabhakaran S Sangha RS Bush RA Long AE Trevick SA. Magnesium, hemostasis, and outcomes in patients with intracerebral hemorrhage. Neurology. (2017) 89:813–9. 10.1212/WNL.0000000000004249 28747450 PMC5580864

[32] Liotta EM Karmarkar A Batra A Kim M Prabhakaran S Naidech AM. Magnesium and hemorrhage volume in patients with aneurysmal subarachnoid hemorrhage. Critical Care Med. (2020) 48:104–10. 10.1097/CCM.0000000000004079 31688193 PMC7008932

[33] Acampa M Camarri S Lazzerini PE Guideri F Tassi R Valenti R. Increased arterial stiffness is an independent risk factor for hemorrhagic transformation in ischemic stroke undergoing thrombolysis. Int J Cardiol. (2017) 243:466–70. 10.1016/j.ijcard.2017.03.129 28747037

[34] Joris PJ Plat J Bakker SJ Mensink RP. Long-term magnesium supplementation improves arterial stiffness in overweight and obese adults: results of a randomized, double-blind, placebo-controlled intervention trial. Am J Clinic Nutri. (2016) 103:1260–6. 10.3945/ajcn.116.131466 27053384

[35] Dong J-f Cruz MA Aboulfatova K Martin C Choi H Bergeron AL. Magnesium maintains endothelial integrity, up-regulates proteolysis of ultra-large von willebrand factor, and reduces platelet aggregation under flow conditions. Thrombosis Haemostasis. (2008) 99:586–93. 10.1160/TH07-11-0694 18327408

[36] Sheu J-R Hsiao G Shen M-Y Fong T-H Chen Y-W Lin C-H. Mechanisms involved in the antiplatelet activity of magnesium in human platelets. Br J Haematol. (2002) 119:1033–41. 10.1046/j.1365-2141.2002.03967.x 12472585

